# Area of Focus in 3D Volumetry and Botulinum Toxin A Injection for Giant Diaphragmatic Hernia with Loss of Domain: A Case Report with Video Illustration

**DOI:** 10.3389/jaws.2024.13448

**Published:** 2024-09-06

**Authors:** Sylvie Nachtergaele, Haitham Khalil, Paul Martre, Jean-Marc Baste, Edouard Roussel

**Affiliations:** ^1^ Department of Digestive Surgery, Cliniques Universitaires St. Luc (UCL), Brussels, Belgium; ^2^ Department of Digestive Surgery, Rouen University Hospital, Rouen, France; ^3^ Department of Digestive Surgery, Hôpital Privé de l’Estuaire, Groupe Ramsay, Le Havre, France; ^4^ Department of General and Thoracic Surgery and INSERM U1096, Rouen University Hospital, Rouen, France

**Keywords:** diaphragmatic hernia, botulinum toxin A, loss of domain, 3D volumetry, case report

## Abstract

**Background:**

Chronic giant diaphragmatic hernia is a severe disease with challenging diagnosis and treatment. Given the risk of loss of domain, the use of botulinum toxin A is an option but has been minimally studied in diaphragmatic hernia surgery.

**Case Report:**

We present a case of a giant diaphragmatic hernia in a 66-years-old patient who showed a 12-year history of progressive chronic respiratory insufficiency. There were not notion of traumatic injuries. The CT-scan showed a giant diaphragmatic hernia with herniation of small bowel, right liver, omentum and transverse colon.

**Method:**

We assessed the risk of loss of domain using a 3D volumetry based on the Sabbagh score and decided to use Botox injection before laparoscopic reduction of the hernia due to the high risk of complications related to the loss of domain. A computed tomography was performed 24 months after surgery and showed no evidence of recurrence. The patient presented an excellent functional result with a normal physical activity.

**Conclusion:**

This report is among the first to highlight the utility of 3D reconstruction in assessing the risk associated with loss of domain and in preparing the abdominal wall with botulinum toxin A for diaphragmatic hernia repair.

## Introduction

Diaphragmatic hernias are a group of disorders characterized by a late diagnosis and often an uncertain origin [1]. They can be congenital, such as Bochdalek hernias, or acquired, typically following trauma, with manifestations potentially emerging years later. This delayed appearance combined with the alternation of respiratory movements, can lead to the development of voluminous hernias accompanied by loss of domain. Although the loss of domain concept is well-documented in ventral hernias, it is seldom discussed in the context of diaphragmatic hernias [2]. Recently, a consensus was reached for ventral hernias, defining them as “large enough such that simple reduction in its contents and primary fascial closure either cannot be achieved without additional reconstructive techniques or cannot be achieved without significant risk of complications due to the raised intra-abdominal pressure” [3].

To assess its risk, two main volumetric definitions were used. The Tanaka method is the ratio of the hernia sac volume (HSV) to the abdominal cavity volume (ACV). The Sabbagh method is the ratio or percentage of the hernia sac volume (HSV) to the total peritoneal volume (TPV = HSV + ACV.) The Sabbagh method is preferred for volume calculation [4], although no consensus exists on the threshold. In the systematic review of Parker, values ranged from 10% to 50%, with the most frequently reported value being 20%. Moreover, our team has described a new tool allowing precise volume calculations through 3D reconstructions, based on the Sabbagh procedure [5].

Preoperative Botulinum Toxin A (BTA) injection has been described as a technique for repairing complex ventral hernias [6]. The relaxation of muscle fibers allows the reintroduction of viscera, potentially decreasing the risk of complications associated with loss of domain. However, the use of BTA in diaphragmatic hernia surgery has been minimally studied.

We present a giant diaphragmatic hernia with herniation of small bowel, right liver, omentum and transverse colon. In our case, the volumetric study confirmed the loss of domain of the hernia content. We considered the injection of Botulinum toxin A useful to avoid an intraperitoneal hyperpressure following the reduction of this voluminous content.

## Case Report

A 66-year-old patient showed a 12-year history of gradually progressive chronic respiratory insufficiency, with no significant history of trauma or accidents. The patient was dependent on long-term oxygen therapy. She was restricted to her home and unable to make any physical effort.

**FIGURE 1:**
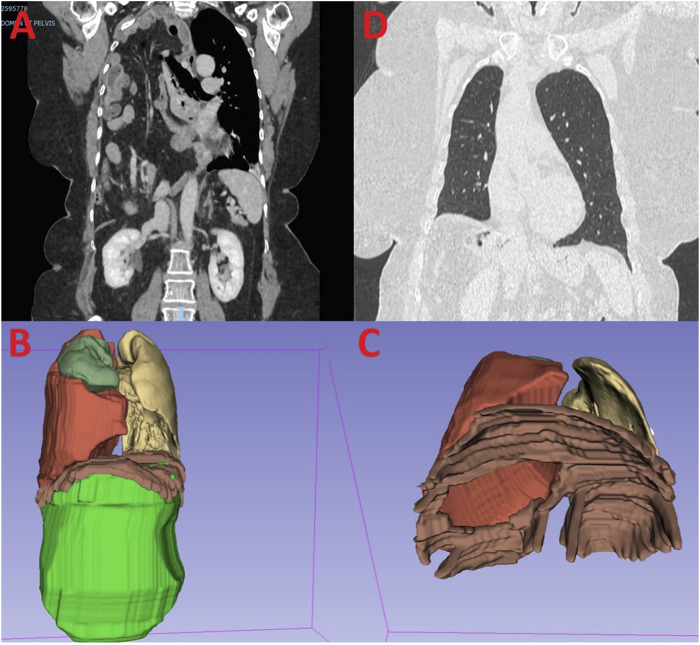
Legend **(A)** Preoperative CT scan showing a giant diaphragmatic hernia. **(B)** 3D reconstruction volumetry with the abdominal volume (green), the hernia volume (red), the right lung (yellow) and the left lung (dark green). **(C)** 3D reconstruction with an inferior view showing the defect through the diaphragm. **(D)** Post-operative CT scan showing no recurrence at 24 months.

A CT scan revealed a large right diaphragmatic hernia with mixed contents including colonic, small bowel, hepatic and omental elements (Figure 1A). Given the size of the hernia, most surgeons refused to operate the patient and eventually, referred her to our centre.

Volumetric analysis using 3D Slicer software showed a hernia sac volume of 2,229 cm³ against an abdominal volume of 3,654 cm³, resulting in a 38% ratio according to the Sabbagh method (Figure 1B). This ratio required preoperative preparation before reducing the hernia contents.

The hernia neck was estimated to be 7 cm in diameter, located at the centre of the diaphragm (Figure 1C).

Bilateral ultrasound-guided identification of the lateral abdominal wall muscles was performed, followed by BTA injections into each muscle at three sites along the upper axillary line (from top to bottom: subcostal, median, anterior superior iliac spine). A total of 16.5 units of BTA were injected per muscle, per site, amounting to 100 units for the entire abdominal wall.

The surgical procedure was scheduled 1 month after the injection. It involved a complete laparoscopic reduction of the hernia sac, diaphragmatic suturing with non-absorbable thread, and reinforcement with 3D Mesh in non-absorbable monofilament polyester with absorbable collagen film (Symbotex™ Composite 15 × 10 cm) secured with absorbable polyester copolymer tackers (AbsorbaTack™). Given the localization of the defect, it couldn’t have been a congenital hernia. A Jackson-Pratt drain and a thoracic drain were placed (Supplementary Video S1).

The patient had an uncomplicated recovery, being discharged on the 12th day without any signs of compartment syndrome. In order to exclude this risk, intra-vesical pressure was monitored daily, as well as oxygen saturation. She suffered at the beginning of hypoventilation, treated by non - invasive ventilation. No recurrence was observed at the 24-month follow-up imaging, with excellent functional results and resolution of dyspnea (Figure 1D).

This video shows the various stages of repair of this giant diaphragmatic hernia and the key points of the operation.

## Discussion

This report is among the first to highlight the utility of 3D reconstruction in assessing the risk associated with loss of domain and in preparing the abdominal wall with BTA for diaphragmatic hernia repair. While the technique of laparoscopic diaphragmatic hernia repair is well-documented, the risk of encountering a loss of domain during these procedures is not well understood [7]. There have been several reports of giant hernias being treated without preoperative optimization [8], whereas others have documented authentic compartment syndrome cases [9].

When the presumed diagnosis shows characteristics of a viscero-abdominal disproportion and surgery is pursued, the surgeon must consider that primary abdominal closure may not be possible and multiple operations may be necessary to correct the defect and achieve closure. Sacrifice of abdominal viscera may also be necessary to reduce the volume of abdominal contents [10].

Abdominal wall surgeons have developed a host of tools to help facilitate fascial closure. BTA is one of the most recently identified treatments and has grown in popularity over recent years, showing great promise in a number of case series and cohort studies [6].

The first described BTA administration into abdominal wall surgery was in 2009, by Ibarra‐Hurtado et al, for the treatment of open abdomen management. In this prospective study, BTA application before abdominal wall hernia repair seems to be useful. The lateral muscles paralysis achieved and transverse hernia defect reduction allows a minimal tension closure thereby reducing postoperative pain and morbidity [11].

The BTA allows a temporary loss of muscle tone, muscle elongation and thinning leading to decrease of intra‐abdominal pressure, avoiding ventral or diaphragmatic hernia recurrence. The use of BTA has already been reported for the treatment of a giant hiatal hernia suggesting its potential benefit in such scenarios [12].

The risk of postoperative abdominal compartment syndrome is defined by the loss of domain calculated with volumetry. Given the 38% ratio, fascial closure could not have been achieved without a significant risk of complications linked to increased intra-abdominal pressure. BTA appears to facilitate fascial closure [13]. Our experience and the data in the literature have led us to adopt this minimally morbid technique, with potentially significant benefits including a demonstrated analgesic effect.

Indeed, the BTA was originally studied to manage post-operative abdominal wall pain. A reduction in pain scores was observed immediately post-operatively and lasted for 3 months of follow-up [14].

We didn’t perform a CT scanner after the BTA injection, as we don't expect a major increase in volume as in a Goni Moreno technique, but rather muscle relaxation [15] difficult to assess by imaging.

Goni Moreno technique is another solution for giant hernias repair through the creation of a progressive preoperative pneumoperitoneum. It allows a high rate of fascial closure [16]. But it wasn’t considered due to the high risk of cardiac compression in case of diaphragmatic hernia.

Access to 3D volumetry is facilitated by numerous reconstruction software programs. We can mention 2 DICOM viewers that can connect to PACS and import segmentations achieved by other applications: Synapse 3D^®^ by Fujifilm and OsiriX by University of Geneva. These programs are both free and open source [17].

The advantage of the method [18] we employed lies in its use of open-source software, using a technique similar to the one we described for ventral hernias, enabling a precise evaluation of both thoracic and abdominal volumes. Our method can be done by any surgeon with basic computer skills and radiological knowledge in an autonomous and a fast manner, thus helping to select the right technique for the right patient [5]. We use it routinely today.

This case report shows no recurrence after 24 months. However, diaphragmatic hernia recurrence may occur years after surgery, and so, future studies with long‐term follow‐up are needed.

One of the limitations of this article is its clinical case nature. It is only an assumption that BTA injection was the best technique to avoid compartmental syndrome in the management of this giant diaphragmatic hernia.

Future controlled trials are warranted to determine the best strategy for avoiding loss of domain and compartment syndrome.

## Conclusion

For this giant diaphragmatic hernia with loss of domain, a pre-operative injection of Botulinum toxin A was crucial in order to decrease the intra‐abdominal pressure and avoid a hernia recurrence.

In view of the risk of loss of domain and therefore of a compartmental syndrome in the context of large diaphragmatic hernia, a detailed volumetric study according to Sabbagh, facilitated by our 3D reconstructions system, seems essential.

This case report underlines the importance of the preoperative work-up, essential before any surgery.

## Patient Perspective

Given the size of the hernia, a lot of surgeons refused to operate. Given the delay in treatment and her chronic respiratory insufficiency, her daily life was severely impacted, with limited physical exertion and dependence on oxygen therapy.

Surgery enabled a rapid and significant improvement in her quality of life, with normal daily physical activity, without needing of oxygen therapy.

## Data Availability

The original contributions presented in the study are included in the article/Supplementary Material, further inquiries can be directed to the corresponding author.
