# Supplementary material for: Area of Focus in 3D Volumetry and Botulinum Toxin A Injection for Giant Diaphragmatic Hernia with Loss of Domain: A Case Report with Video Illustration
Source: J Abdom Wall Surg. 2024 Sep 6;3:13448. doi: 10.3389/jaws.2024.13448 (PMC11412849; doi:10.3389/jaws.2024.13448)
Supplement: Supplementary file 1 [file DataSheet1.PDF]

Area of focus in 3D Volumetry and Botulinum Toxin A Injection for Giant Diaphragmatic Hernia with Loss of Domain : A Case Report with Video Illustration

Authors

* Sylvie Nachtergaele M.D.[1], Haitham Khalil M.D. [2], Paul Martre M.D. [3], Jean-Marc Baste M.D. Ph.D. [4] , Edouard Roussel M.D.[2]

Description

This video shows the various stages of repair of this giant diaphragmatic hernia with loss of domain : the preoperative injection of Botulinum toxin A and the key points of the laparoscopic surgery.

Link to the video

https://drive.google.com/file/d/11LD1g2GYU311Jm0TPrPaR-ZXXmRLT6Dc/view?usp=sharing
